# ﻿*Hedysarumqilianshanense* sp. nov. (Fabaceae, Hedysareae), a new species from the Qilianshan Mountains in Gansu, China

**DOI:** 10.3897/phytokeys.237.116236

**Published:** 2024-01-22

**Authors:** Pei-Liang Liu, Qian-Xi Guo, Jian-Qi Zhang, Lu-Lu Xun, Yuan Lu, Ming Yue

**Affiliations:** 1 Key Laboratory of Resource Biology and Biotechnology in Western China, Ministry of Education, Northwest University, Xi’an, Shaanxi 710069, China Northwest University Xi’an China; 2 Linhai City Natural Resources and Planning Bureau of Zhejiang Province, Linhai, Zhejiang 317099, China Linhai City Natural Resources and Planning Bureau of Zhejiang Province Linhai China; 3 Shaanxi Engineering Research Centre for Conservation and Utilization of Botanical Resources, Xi’an Botanical Garden of Shaanxi Province (Institute of Botany of Shaanxi Province), Xi’an, Shaanxi 710061, China Xi’an Botanical Garden of Shaanxi Province Xi’an China

**Keywords:** Karyotype, phylogeny, Qinghai-Tibetan Plateau, taxonomy

## Abstract

*Hedysarumqilianshanense***sp. nov.** (Fabaceae, Hedysareae) is described and illustrated from the Qilianshan Mountains in Gansu, China. This new species is similar to *H.przewalskii*, but can be distinguished by its corolla being light purple to purple, standard 15–19 mm long, wings 14–16 mm long, keels 16–19 mm long, and the ovary and legume being glabrous. The new species can be easily distinguished from *H.neglectum* Ledeb. by its bract being shorter than the pedicel, and the ovary and legume being glabrous. Phylogenetic tree based on the nuclear ITS and ETS sequences shows that *H.qilianshanense* is sister to *H.przewalskii*, while the tree based on the plastid *psbA-trnH*, *trnC-petN*, *trnL-F*, *trnS-G* and *petN-psbM* sequences shows *H.qilianshanense* as sister to a clade consisting of *H.hedysaroides*, *H.inundatum*, *H.americanum* and *H.neglectum*. The new species is a diploid with the chromosome number *2n* = 14. Based on morphological, phylogenetic and karyotypic evidence, the new species may originate from an ancient homoploid hybrid speciation event.

## ﻿Introduction

The genus *Hedysarum* L. (Fabaceae, Hedysareae) consists of more than 160 species, widely distributed in temperate Asia, Europe, northern Africa and North America (Xu and Choi 2010). Previous phylogenetic analyses delimited *Hedysarum* into three sections, *i.e.*, H.sect.Hedysarum, H.sect.Stracheya (Benth.) B. H. Choi & H. Ohashi, and H.sect.Multicaulia (Boiss.) B. Fedtsch. (Duan et al. 2015; Liu et al. 2017b, 2019; Nafisi et al. 2019). Species of H.sect.Hedysarum mostly inhabit temperate forests, alpine meadows and arctic tundra in Eurasia and North America (Xu and Choi 2010; Duan et al. 2015).

The Qinghai-Tibetan Plateau (QTP) is the largest and highest plateau on the earth. More than 12,000 species of vascular plants inhabit in QTP and such species richness also exhibit a high level of endemism (Wen et al. 2014). The QTP harbors about 24 species of H.sect.Hedysarum, with 22 species endemic to the QTP. Most of these species are distributed in the Himalayas on the southern border of the QTP, and the Hengduan Mountains on the eastern border of the QTP (Ohashi and Tateishi 1975; Xu and Choi 2010; Choi et al. 2011; Liu et al. 2017a). The Qilianshan Mountains are a huge mountain range that lie on the northeastern border of the QTP, separating the QTP from the deserts in the north. Only one species, *H.algidum* L. Z. Shue, is known from the Qilianshan Mountains. Species diversity in the Qilianshan Mountains remains to be further explored.

When the first author was examining specimens of *Hedysarum* deposited in the herbarium of Northwest Normal University (NWTC), a specimen of H.sect.Hedysarum collected from the Qilianshan Mountains was found to be different from any of the described species. Field expeditions to the Qilianshan Mountains discovered additional populations of this form and subsequent morphological and phylogenetic analyses indicated that they represent a new species that is described in the present paper.

## ﻿Materials and methods

### ﻿Taxon sampling

Samples were collected from three populations of the putative new species, including six individuals from the Xiaogushan population, six individuals from the Wulin’gou population, and five individuals from the Sidalong population. Each of these samples were sequenced for phylogenetic analyses. Other 23 species in H.sect.Hedysarum were selected to test the phylogenetic position of the putative new species. Species in H.sect.Stracheya were selected for outgroup comparison because previous studies showed that H.sect.Stracheya is sister to H.sect.Hedysarum (Liu et al. 2017a, 2017b, 2019). Voucher information is given in the Appendix 1.

### ﻿DNA extraction, PCR and sequencing

Silica-gel dried leaves were used to extract genomic DNA by using the Qiagen DNeasy® Plant Mini Kit (Hilden, Germany). The nuclear ribosomal external transcribed spacer (ETS) and internal transcribed spacer (ITS), and the plastid *psbA-trnH*, *trnC-petN*, *trnL-F*, *trnS-G* and *petN-psbM* sequences were amplified using Polymerase chain reaction (PCR). Primers and PCR conditions followed the previous paper (Liu et al. 2017b). Amplicons were sequenced in both directions using the amplification primers. All sequences were deposited in GenBank and the accession numbers are provided in Appendix 1.

### ﻿Phylogenetic analysis

Phylogenetic trees were reconstructed based on the newly generated sequences together with the previously published data (Duan et al. 2015; Liu et al. 2017a, 2017b, 2019). MUSCLE (Edgar 2004) implemented in Geneious v.9 (Kearse et al. 2012) was used to conduct multiple sequence alignments. The best-fit nucleotide substitution model was determined by jModelTest v.2.1.7 (Darriba et al. 2012). A GTR + G model was applied to the combined nuclear data, and a GTR + G + I model was applied to the combined plastid data. Phylogenetic trees were constructed based on the nuclear and the plastid data separately because of the phylogenetic incongruence between the nuclear and the plastid trees (see results). Bayesian inferences (BI) were conducted in MrBayes v.3.2.5 (Ronquist and Huelsenbeck 2003; Ronquist et al. 2012). We ran BI for 10,000,000 generations, and trees were sampled every 1,000 generations. The first 2,500 trees were discarded, and the remaining trees were used to build a 50% majority-rule consensus tree with posterior probabilities (PP). The maximum likelihood (ML) and maximum parsimony (MP) analyses were conducted using RAxML v.8.2 (Stamatakis 2014) and PAUP* 4.0a169 (Swofford 2002), respectively. The ML and MP bootstrap analyses were each performed with 1,000 replicates. Bootstrap support values (BML, BMP) from the ML and MP analyses were labeled on the corresponding branches of the BI trees.

### ﻿Chromosome number count

One available seed of the putative new species (voucher: *P. L. Liu 458*, see Appendix 1 for details) was germinated in a culture dish with wet filter paper at room temperature. When root grew to ca. 5 mm long, it was treated in 2 mmol·L^-1^ 8-hydroxyquinoline solution at room temperature for 4 h. It was then fixed with a mixture of acetic acid and ethyl alcohol (1:3 volume) at 4 °C and stored overnight. The root tissue was digested with 1 mol·L^-1^ hydrochloric acid at 60 °C for 3 min, and cleaned thoroughly with tap water. The root tip was stained with carbol fuchsin and squashed on a glass slide. Well-spread mitotic metaphase chromosomes were examined and photographed with 100× oil lens on a Nikon Eclipse 55i microscope.

## ﻿Results

### ﻿Nuclear data

**Figure 1.**
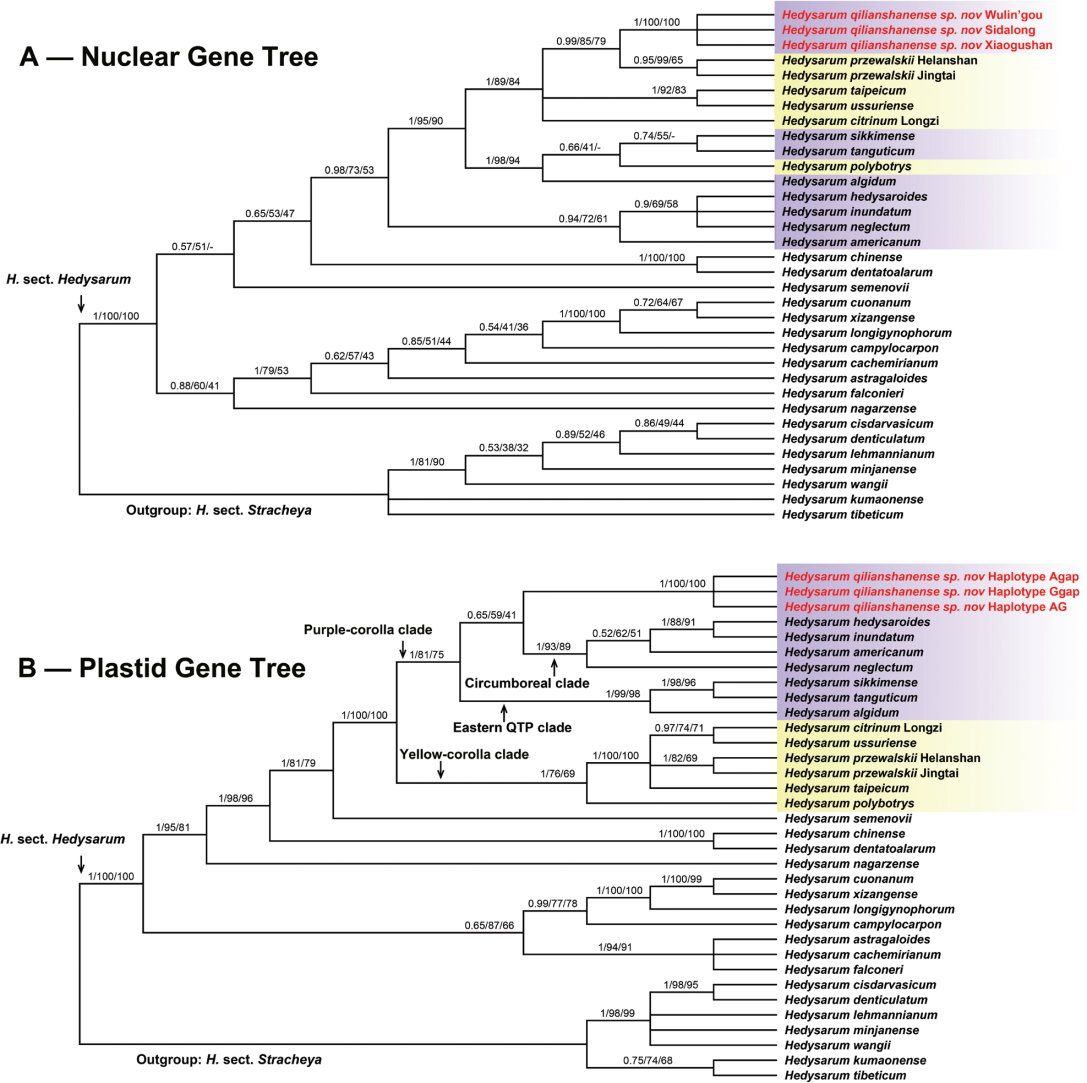
Bayesian trees based on the combined nuclear ETS and ITS sequences (**A**) and the combined plastid *psbA-trnH*, *trnC-petN*, *trnL-F*, *trnS-G* and *petN-psbM* sequences (**B**). The Bayesian posterior probabilities (PP), the maximum likelihood and the maximum parsimony bootstrap supports (BML, BMP) are above the branches. A dash indicates a branch that is not found in the maximum parsimony tree. The purple and yellow shades on taxa names indicates corolla colors.

The 17 individuals from three populations (Xiaogushan, Wulin’gou and Sidalong) of the putative new species had identical ETS and ITS sequences. The nuclear phylogenetic tree based on the combined ETS and ITS sequences (Fig. 1A) shows the putative new species as sister to *H.przewalskii* Yakovlev (PP = 0.99, BML = 85%, BMP = 79%). These two species formed a clade with *H.taipeicum* (Hand.-Mazz.) K. T. Fu, *H.ussuriense* I. Schischkin & Kom. and *H.citrinum* E. G. Baker (PP = 1, BML = 89%, BMP = 84%).

### ﻿Plastid data

Three plastid haplotypes, namely AG, Agap and Ggap (Table 1), were found from the combined plastid *psbA-trnH*, *trnC-petN*, *trnL-F*, *trnS-G* and *petN-psbM* sequences of the 17 individuals from three populations of the new species. The sequence variations of the haplotypes and the distribution of the haplotypes in populations and individuals were showed in Table 1. Therefore, each of these three haplotypes were included in the phylogenetic analyses. In the plastid tree based on the combined *psbA-trnH*, *trnC-petN*, *trnL-F*, *trnS-G* and *petN-psbM* sequences (Fig. 1B), the new species was weakly supported (PP = 0.65, BML = 59%, BMP = 41%) to be sister to the circumboreal clade (comprising *H.neglectum* Ledeb., *H.americanum* (Michx.) Britton, *H.inundatum* Turcz. and *H.hedysaroides* Schinz & Thell.). The new species plus the circumboreal clade was sister to the eastern QTP clade (comprising *H.algidum* L. Z. Shue, *H.tanguticum* B. Fedtsch. and *H.sikkimense* Benth. ex Baker), and these clades formed the purple-corolla clade (PP = 1, BML = 81%, BMP = 75%). The purple-corolla clade was sister to (PP = 1, BML = 100%, BMP = 100%) the yellow-corolla clade (comprising *H.polybotrys* Hand.-Mazz., *H.taipeicum*, *H.przewalskii*, *H.ussuriense* and *H.citrinum*).

**Table 1.** Haplotypes from the combined plastid *psbA-trnH*, *trnC-petN*, *trnL-F*, *trnS-G* and *petN-psbM* sequences of *Hedysarumqilianshanense*.

Haplotype name	Sequence variation					Distribution in populations (number of individuals)
	*psbA-trnH*	*trnC-petN*	*trnL-F*	*trnS-G*	*petN-psbM*	
AG	A	G	identical	identical	identical	Xiaogushan (6), Wulin’gou (4)
Agap	A	gap	identical	identical	identical	Wulin’gou (2), Sidalong (4)
Ggap	G	gap	identical	identical	identical	Sidalong (1)

### ﻿Chromosome number count

**Figure 2.**
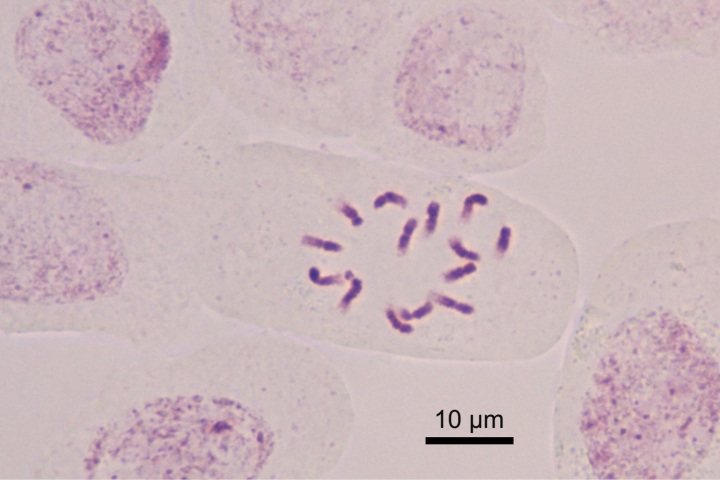
Mitotic metaphase chromosomes from root tip of *Hedysarumqilianshanense*.

A total of 21 cells with well-spread mitotic metaphase chromosomes were observed. All cells showed that the chromosome number of the new species was *2n* = 14 (Fig. 2).

### ﻿Taxonomy

#### ﻿ Hedysarum qilianshanense

Taxon classificationPlantaeFabalesFabaceae

P.L.Liu, sp. nov. (H. sect. Hedysarum)

387B25F9-6890-5EEE-A2B3-C64D70C478A0

urn:lsid:ipni.org:names:77334725-1

Figs 3 , 4

##### Type.

China, Gansu Province, Su’nan County, the Heihe River valley, Xiaogushan, in crevice on stony slope, 2053 m above sea level (a. s. l.), 38°41′6.38″N, 110°3′9.98″E, 21 June 2019, *P. L. Liu 458* (Holotype, WUK!, barcode WUK0536471; Isotypes, WUK!, barcodes WUK0536466-WUK0536470, WNU!).

##### Diagnosis.

This new species is morphologically similar to *H.przewalskii*, but can be distinguished by its light purple to purple corolla (vs. light yellow to yellow corolla), 15–19 mm long standard (vs. 10–14 mm long standard), 14–16 mm long wings (vs. 10–14 mm long wings), 16–19 mm long keels (vs. 12–17 mm long keels), and glabrous ovaries and legumes (vs. often pubescent, sometimes glabrate or glabrous ovaries and legumes). The new species can be easily distinguished from *H.neglectum* by its bract shorter than pedicel (vs. bract longer than pedicel), and glabrous ovaries and legumes (vs. pubescent ovaries and legumes) (Table 2).

**Figure 3.**
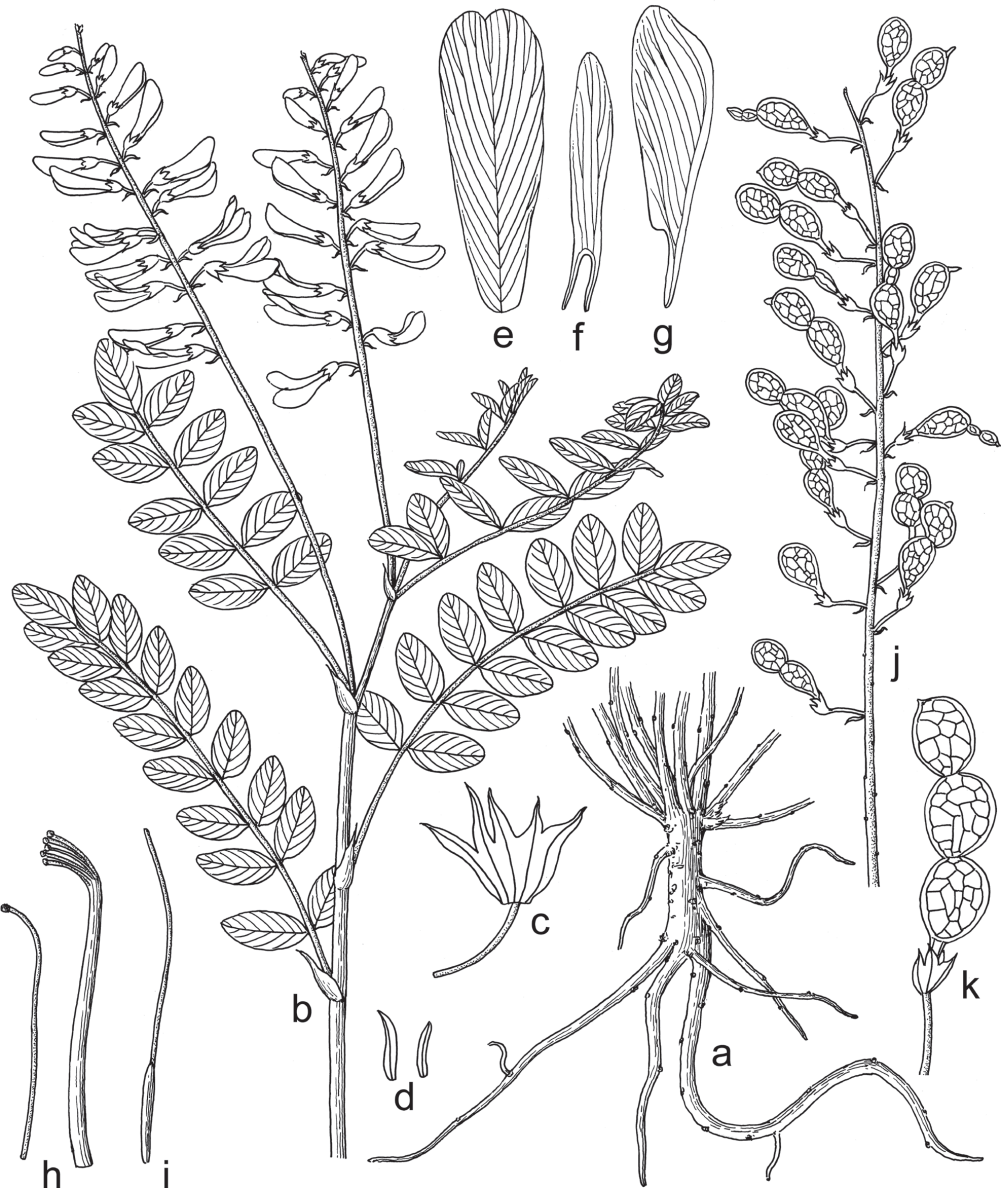
Illustration of *Hedysarumqilianshanense***a** root and basal part of stems **b** upper part of plant **c** calyx tube (split between an adaxial tooth and a lateral tooth) **d** bracteoles **e** standard **f** wing **g** keel **h** androecium **i** pistil **j** infructescence **k** legume. Drawn by Xiu-Zhen Wu.

**Table 2.** Morphological comparison of *Hedysarumqilianshanense*, *H.przewalskii* and *H.neglectum*.

	*H.qilianshanense*	*H.przewalskii*	*H.neglectum*
Bract	shorter than pedicel	shorter than pedicel	longer than pedicel
Corolla color	light purple to purple	light yellow to yellow	purple
Standard length	15–19 mm	10–14 mm	13–14 mm
Wing length	14–16 mm	10–14 mm	13–14 mm
Keel length	16–19 mm	12–17 mm	15–16 mm
Ovary and legume	glabrous	often pubescent, sometimes glabrate or glabrous	pubescent

##### Description.

Perennial herbs, 30–100 cm tall. Main root stout, woody, up to 1.3 cm in diameter. Stems cespitose, ascending, branched; internodes glabrous or loosely pubescent, nodes pubescent. Leaves imparipinnate, alternate, 12–30 cm long; stipules connate, opposite to leaves, wide triangular, membranous, brown, glabrous, apex shallowly bilobed, lower ones 8–17 mm long, becoming smaller in upper part of stem; rachises sulcate, glabrous or sparsely pubescent; leaflets 9–19, opposite or alternate; petiolules ca. 1 mm long, pubescent; leaflet blades elliptic, ovate-elliptic, oblong, 12–40 × 7–25 mm, adaxial surface glabrous, abaxial surface sparsely pubescent along midvein, base wide cuneate or rounded, apex obtuse, rounded or retuse. Racemes axillary, exceeding leaves, 15–42 cm long, with 15–50 flowers, peduncles pubescent; pedicel 3–6 mm long, pubescent; bracts linear, with brown midvein, pubescent, 2–5 mm long; bracteoles 2, linear, with brown midvein, pubescent, 2–3.5 mm long; calyx tube campanulate, 3–4 mm long, pubescent; calyx teeth 5, pubescent, the two adaxial teeth triangular, ca. 1 mm long, the two lateral teeth narrowly triangular, 1.5–2.5 mm long, the abaxial tooth linear-triangular, 2–3 mm long; corolla light purple to purple; standard obovate, 15–19 × 5.5–7 mm, apex retuse, base attenuate; wings 14–16 × 2–2.5 mm, auricle linear, as long as claw, 2–3 mm long; keels 16–19 × 4–5 mm, auricle triangular, ca. 1 mm long; androecium diadelphous, 12–17 mm long; ovary linear, glabrous, style ca. 13 mm long. Legume a loment, divided into 2–4 articles, with a small beak at apex; articles elliptic, compressed, 8–10 mm × 6–7 mm, glabrous, with reticulate veins, with a narrow wing ca. 0.5 mm wide along the dorsal suture only. Seed reniform, yellow, ca. 3 × 2 mm.

**Figure 4.**
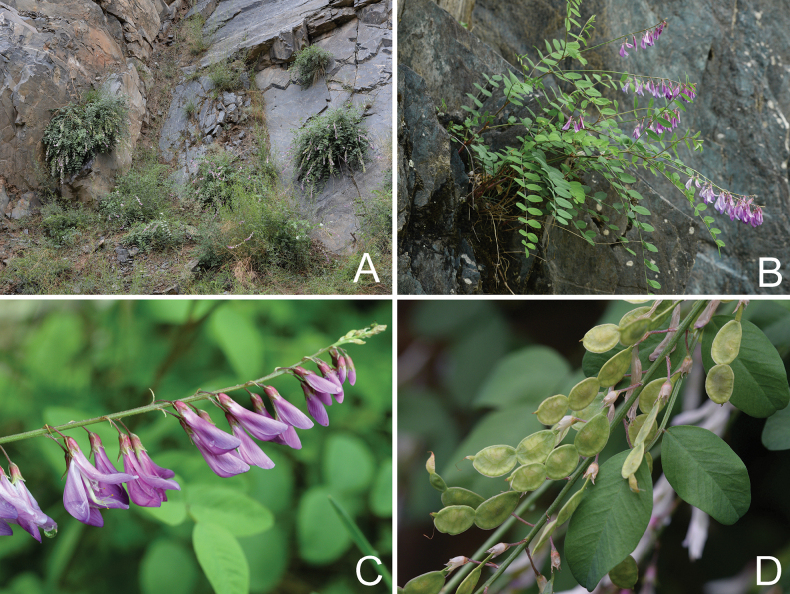
Photos of *Hedysarumqilianshanense* from the field **A** habitat **B** plant **C** raceme **D** infructescence. Photographed by Pei-Liang Liu.

##### Phenology.

Flowering and fruiting in June.

##### Distribution and habitat.

*Hedysarumqilianshanense* is only known from Su’nan, Gansu, China. It grows in stony slope and forest edge in valley, 2053–3000 m a. s. l.

##### Etymology.

The epithet *qilianshanense* is transliterated from the type location, Qilianshan Mountains in China. The Chinese vernacular name for this new species is 祁连山岩黄耆 (qí lián shān yán huáng qí).

##### Other specimens examined (Paratypes).

China, Gansu Province, Su’nan County, Sidalong, Wulin’gou, on stony slope, 3000 m a. s. l., 21 June 1986, *Sheng Huan Bao Dui 86055* (NWTC!); Su’nan County, Sidalong, Wulin’gou, in crevice on stony slope, 2542 m a. s. l., 38°28′1.67″N, 99°56′54.63″E, 21 June 2019, *P. L. Liu 461* (WUK!, barcodes WUK0536462, WUK0536463, WNU!); Su’nan County, Sidalong, on slope on forest edge, 2632 m a. s. l., 38°27′26.6″N, 99°54′52.69″E, 21 June 2019, *P. L. Liu 470* (WUK!, barcodes WUK0536464, WUK0536465, WNU!).

## ﻿Discussion

**Figure 5.**
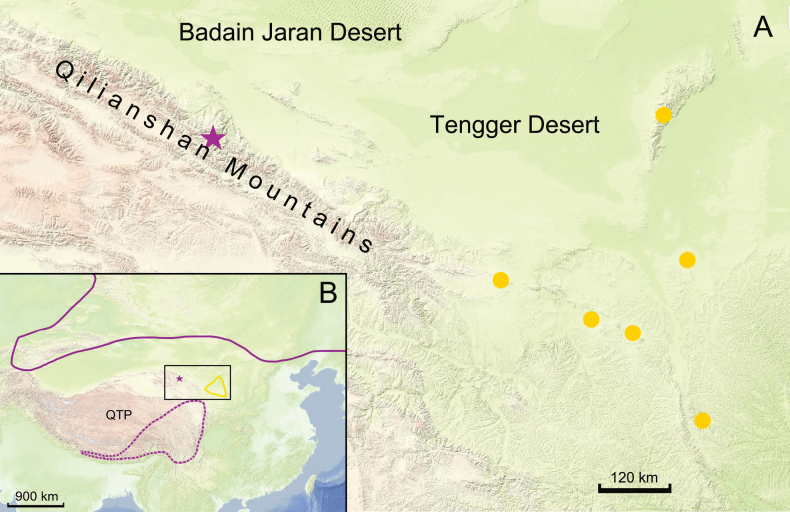
Distributions of *Hedysarumqilianshanense* and related species and clades. The purple star represents *H.qilianshanense*; yellow dots and the area surrounded by yellow line represent *H.przewalskii*; the area surrounded by the purple line represent the circumboreal clade; the area surrounded by the purple dotted line represent the eastern QTP clade. Purple or yellow indicates the corolla color. The black frame in Fig. 5B is enlarged in Fig. 5A. Maps from shaanxi.tianditu.gov.cn.

The phylogenetic positions of *H.qilianshanense* are different in the nuclear and plastid trees. *Hedysarumqilianshanense* and *H.przewalskii* are similar to each other in well-developed stem and large, elliptic, ovate-elliptic or oblong leaflets. On the other hand, *H.qilianshanense* is clearly different from *H.przewalskii* in flower and fruit features (Table 2). *Hedysarumqilianshanense* is distributed in the central part of the Qilianshan Mountains, whereas *H.przewalskii* is distributed east of the Qilianshan Mountains (Fig. 5). Thus, these two species are isolated from each other. The Badain Jaran Desert and the Tengger Desert may serve as geographic barriers between the two species because they are both mesophytes inhabiting mountainous regions.

In the plastid tree, however, *H.qilianshanense* is a member of the purple-corolla clade (Fig. 1B). All species in this clade have a purple corolla, but are diversified in their morphology of the stem, leaf, flower and fruit. Species of the circumboreal clade are distributed in northern China and Siberia (*H.inundatum* and *H.neglectum*), Europe (*H.hedysaroides*) and North America (*H.americanum*). Species of the eastern QTP clade (*H.sikkimense*, *H.tanguticum* and *H.algidum*) are distributed in the eastern Himalayas and the Hengduan Mountains. Thus, *H.qilianshanense* is isolated from other species of the purple-corolla clade (Fig. 5).

The incongruent position of *H.qilianshanense* in the nuclear and plastid gene trees indicates that *H.qilianshanense* may have originated from a hybridization event. The nuclear gene tree tracks one potential parent, probably an ancestor of *H.przewalskii*, and the plastid gene tree tracks the other potential parent, probably an ancestor in the purple-corolla clade. Morphologically, *H.qilianshanense* has similar stem, leaf and leaflet features as *H.przewalskii*, and the same corolla color with species of the purple-corolla clade.

A previous study (Choi and Ohashi 2003) concluded that the basic chromosome number of H.sect.Hedysarum is *x* = 7. Therefore, *H.qilianshanense* is most likely a diploid although chromosome pairing was not observed. *Hedysarumprzewalskii* was also reported as diploid, *2n* = 14 [Yan et al. 1995, reported for H.polybotrysvar.alaschanicum (B. Fedtsch.) H. C. Fu & Z. Y. Chu, a synonym of *H.przewalskii*]. In the purple-corolla clade, *H.hedysaroides*, *H.americanum*, *H.neglectum*, *H.sikkimense* and *H.tanguticum* were also reported as diploid, *2n* = 14, (Löve 1979, 1985; Yurkevich et al. 2021), and only *H.inundatum* was reported as tetraploid, *2n* = 28 (Löve 1981). The chromosome number of *H.algidum* is unknown.

In conclusion, based on the morphological, phylogenetic and karyotypic evidences, *H.qilianshanense* may have originated from homoploid hybrid speciation. Because of the allopatric distribution of *H.qilianshanense*, *H.przewalskii*, the circumboreal clade and the eastern QTP clade, the hybrid speciation is most likely to be an ancient, rather than a recent, event.

## Supplementary Material

XML Treatment for Hedysarum qilianshanense

## ﻿Additional information

### Author ORCIDs

Pei-Liang Liu https://orcid.org/0000-0001-6566-7824

Qian-Xi Guo https://orcid.org/0009-0002-9144-5752

Lu-Lu Xun https://orcid.org/0000-0002-6861-5883

Yuan Lu https://orcid.org/0000-0002-0329-0803

Ming Yue https://orcid.org/0000-0002-8618-1126

### Data availability

The newly generated DNA sequences have been deposited in GenBank and the accession numbers can be found in Appendix 1.

## ﻿Appendix 1

Taxon name, geographical locality, voucher, herbarium code, and GenBank accession number for the sequences used in this study. For each sample, accession numbers are given for the ETS, ITS, *psbA-trnH*, *trnC-petN*, *trnL-F*, *trnS-G* and *petN-psbM* sequences. A dash (–) indicates a missing sequence. New sequences generated in this study are indicated by an asterisk (*).

*Hedysarumalgidum* L. Z. Shue: China, Gansu, W. Q. Yang 2008010 (WUK), KY365837, KP338149, KP338400, KY366037, KP338272, KY365888, KY365987;

*Hedysarumamericanum* (Michx.) Britton: Canada, Tuktut Nogait National Park, L. J. Gillespie et al. 8934 (US), KY365838, KP338150, KP338402, KY366038, KY366134, KY365889, KY365988;

*Hedysarumastragaloides* Benth. ex Baker: Pakistan, Punjab, W. Koelz 5024 (US), KY367282, KP338153, KP338405, KY367325, KP338275, OR971769*, KY367309;

*Hedysarumcachemirianum* Benth. ex Baker: Kashmir, Kishenganga Valley and the road to Nanga Parbat, R.R. & I.D. Stewart 18354 (US), KY367283, KY367300, KY367343, KY367326, OR971763*, –, KY367310;

*Hedysarumcampylocarpon* H. Ohashi: China, Xizang, Jilong, Z.Y. Chang et al. 2013073 (WUK), KY367284, KY367301, KY367344, KY367327, –, –, KY367311;

*Hedysarumcampylocarpon* H. Ohashi: China, Xizang, Nielamu, Z.Y. Chang et al. 2011203 (WUK), –, –, –, –, KP338279, OR971765*, –;

*Hedysarumchinense* (B. Fedtsch.) Hand.-Mazz.: China, Shaanxi, Ningshan, Z. M. Jiang 1565 (WUK), KY365842, KP338159, KP338412, KY366042, KP338280, KY365891, KY365992;

*Hedysarumcisdarvasicum* Kamelin & Karimova: Tajikistan, Darvaz, Yakhsu river. basin, R. Kamelin s. n. (TAD), MK639303, MK639233, MK639289, –, MK639275, MK639261, MK639247;

*Hedysarumcitrinum* E. G. Baker: China, Xizang, Longzi, Y. S. Chen et al. 13-468. (WUK), OR971731*, OR982385*, OR971741*, OR971748*, OR971757*, OR971778*, –;

*Hedysarumcuonanum* P. L. Liu, J. Wen & Zhao Y. Chang: China, Xizang, Cuona, Y. S. Chen et al. 13-0948 (WUK), KY367286, KY367302, KY367345, KY367329, OR971764*, OR971767*, KY367312;

*Hedysarumdentatoalatum* K. T. Fu: China, Henna, Luanchuan, Z. Y. Chang et al. 2013267 (WUK), KY365844, KP338162, KP338415, KY366044, KP338283, KY365893, KY365994;

*Hedysarumdenticulatum* Regel: Kyrgyzstan, Osh, Alay Valley, I. Sodombekov & N. Rogova KPL_00816 (MO), KY365845, KY366156, KY365760, –, KY366137, KY365894, KY365995;

*Hedysarumfalconeri* Baker, Pakistan, Karakoram, O. Polunin 6096 (F), KY367287, KP338163, KP338416, KY367330, KP338284, OR971768*, KY367313;

*Hedysarumhedysaroides* Schinz & Thell.: Russia, Kamchatka, Tolbachik Volcano, S. McDonald & N. A. Brummitt 23 (US), KY365847, KP338168, KP338421, KY366046, KP338288, KY365896, KY365997;

*Hedysaruminundatum* Turcz.: China, Shanxi, Huangtudui 01313 (WUK), KY365848, KP338170, KP338423, KY366047, KP338290, KY365897, KY365998;

*Hedysarumkumaonense* Benth. ex Baker: China, Xizang, Jilong, Z. Y. Chang et al. 2013084 (WUK), KY367288, KP338174, KP338427, KY367331, KP338294, KY365899, KY367314;

*Hedysarumlehmannianum* Bunge: Tajikistan, Zeravshan range, Pastrud-darya river. basin, Turzun, Abdusalyamova, Zhogoleva & Ovchinnikov 4932 (TAD), MK639308, MK639238, MK639294, –, MK639280, MK639266, MK639252;

*Hedysarumlongigynophorum* C. C. Ni: China, Xizang, Gongbujiangda, Z. Y. Chang. et al. QZ620 (WUK), KY367290, KP338175, KP338428, KY367333, KP338295, KY365900, KY367316;

*Hedysarumminjanense* Rech. f.: China, Xinjiang, Tashikuergan, Xizhixinjiangdui. 1091 (WUK), KY365852, KY366159, KY365763, –, KY366140, KY365902, KY366001;

*Hedysarumnagarzense* C. C. Ni: China, Xizang, Langkazi, L.R. Xu 1463 (WUK), KY367292, KY367305, KY367348, KY367335, OR971762*, OR971770*, KY367318;

*Hedysarumneglectum* Ledeb.: China, Xinjiang, Xinyuan, L. R. Xu 1533 (WUK), KY365853, KY366160, KY365764, KY366050, KY366141, KY365903, KY366002;

*Hedysarumpolybotrys* Hand.-Mazz.: China, Gansu, Xiahe, Z. Y. Chang et al. QZ042. (WUK), KY365854, KP338182, KP338434, KY366051, KP338300, KY365905, KY366003;

*Hedysarumprzewalskii* Yakovlev (Helanshan): China, Ningxia, Helanshan, R. B. Zhu. s. n. (WUK), OR971730*, OR982389*, OR971740*, OR971750*, OR971760*, OR971776*, –;

*Hedysarumprzewalskii* Yakovlev (Jingtai): China, Gansu, Jingtai, Z. Y. Yu & Y. P. Xu 3255 (WUK), OR971729*, OR982390*, –, OR971751*, OR971761*, OR971775*, –;

*Hedysarumqilianshanense* P. L. Liu (Sidalong or Haplotype Ggap): China, Gansu, Su’nan, Sidalong, P. L. Liu 470 (WUK and WNU), OR971724*, OR982386*, OR971737*, OR971746*, OR971755*, OR971772*, OR971732*;

*Hedysarumqilianshanense* P. L. Liu (Wulin’gou or Haplotype Agap): China, Gansu, Su’nan, Wulin’gou, P. L. Liu 461 (WUK and WNU), OR971725*, OR982387*, OR971738*, OR971747*, OR971754*, OR971774*, OR971733*;

*Hedysarumqilianshanense* P. L. Liu (Xiaogushan or Haplotype AG): China, Gansu, Su’nan, Xiaogushan, P. L. Liu 458 (WUK and WNU), OR971726*, OR982388*, OR971739*, OR971745*, OR971756*, OR971773*, OR971734*;

*Hedysarumsemenowii* Regel & Herder: Kazakhstan, Ulken, I. Roldugin 4823 (US), KY365856, KP338183, KP338435, KY366053, KP338301, KY365907, KY366005;

*Hedysarumsikkimense* Benth. ex Baker: China, Xizang, Yadong Y. S. Chen et al. 13-1772 (WUK), KY367294, KY367306, KY367349, KY367337, OR971753*, OR971771*, KY367320;

*Hedysarumtaipeicum* (Hand.-Mazz.) K. T. Fu: China, Shaanxi, Mt. Taibai, P. L. Liu. 681-1 (WNU), OR971727*, OR982391*, OR971742*, OR971752*, OR971759*, OR971777*, OR971736*;

*Hedysarumtanguticum* B. Fedtsch.: China, Qinghai, Chengduo, Z. Y. Chang et al. 2010230 (WUK), KY365857, KP338188, KP338440, KY366056, KP338306, KY365910, KY366008;

*Hedysarumtibeticum* (Benth.) B. H. Choi & H. Ohashi: China, Xizang, Langkazi, Z. Y. Chang et al. 2011111 (WUK), KY367296, KP338189, KP338441, KY367339, KP338307, KY365911, KY367321;

*Hedysarumussuriense* I. Schischkin & Kom.: China, Jilin, Mt. Changbai, M. Z. Sun. s. n. (WNU), OR971728*, OR982384*, OR971743*, OR971749*, OR971758*, –, OR971735*;

*Hedysarumwangii* P. L. Liu & Zhao Y. Chang: China, Gansu, Xiahe, Qu’ao, P. L. Liu. 432 (WUK and WNU), MK639312, MK639242, MK639298, OR971744*, MK639284, MK639270, MK639256;

*Hedysarumxizangense* C. C. Ni: China, Xizang, Longzi, Sananqulin, Y. S. Chen et al. 13-0886 (WUK), KY367297, KY367307, KY367350, KY367340, KP338310, OR971766*, KY367322.
